# Plasma metabolite profiles associated with the World Cancer Research Fund/American Institute for Cancer Research lifestyle score and future risk of cardiovascular disease and type 2 diabetes

**DOI:** 10.1186/s12933-023-01912-6

**Published:** 2023-09-16

**Authors:** Santiago Rios, Jesús F. García-Gavilán, Nancy Babio, Indira Paz-Graniel, Miguel Ruiz-Canela, Liming Liang, Clary B Clish, Estefania Toledo, Dolores Corella, Ramón Estruch, Emilio Ros, Montserrat Fitó, Fernando Arós, Miquel Fiol, Marta Guasch-Ferré, José M Santos-Lozano, Jun Li, Cristina Razquin, Miguel Ángel Martínez-González, Frank B Hu, Jordi Salas-Salvadó

**Affiliations:** 1 Universitat Rovira i Virgili, Departament de Bioquímica i Biotecnologia, Alimentaciò, Nutrició Desenvolupament i Salut Mental ANUT-DSM, Reus, Spain; 2 CIBER de Fisiopatología de la Obesidad y Nutrición, Instituto de Salud Carlos III, Madrid, Spain; 3 Institut d’Investigació Sanitària Pere Virgili (IISPV), Reus, Spain; 4 Department of Preventive Medicine and Public Health, University of Navarra, IdiSNA, Pamplona, Spain; 5 Department of Epidemiology, Harvard T.H. Chan School of Public Health, Boston, MA USA; 6 Department of Biostatistics, Harvard T.H. Chan School of Public Health, Boston, MA USA; 7 Broad Institute of MIT and Harvard, Cambridge, MA USA; 8 Department of Preventive Medicine, University of Valencia, Valencia, Spain; 9 Department of Internal Medicine, Institut d’Investigacions Biomèdiques August Pi Sunyer (IDIBAPS), Hospital Clínic, Barcelona, Spain; 10 Department of Endocrinology and Nutrition, Lipid Clinic, Institut d’Investigacions Biomèdiques August Pi Sunyer (IDIBAPS), Hospital Clínic, Barcelona, Spain; 11 Unit of Cardiovascular Risk and Nutrition, Institut Hospital del Mar de Investigaciones Médicas Municipal d’Investigació Médica (IMIM), Barcelona, Spain; 12 Department of Cardiology, Hospital Universitario de Álava, Vitoria, Spain; 13 Health Research Institute of the Balearic Islands (IdISBa), Hospital Son Espases, Palma de Mallorca, Spain; 14 Department of Nutrition, Harvard T.H. Chan School of Public Health, Boston, MA USA; 15 Department of Public Health and Novo Nordisk Foundation Center for Basic Metabolic Research, Faculty of Health and Medical Sciences, University of Copenhagen, Copenhagen, Denmark; 16 Research Unit, Department of Family Medicine, Distrito Sanitario Atención Primaria Sevilla, Sevilla, Spain; 17 Channing Division of Network Medicine, Department of Medicine, Brigham and Women’s Hospital, Harvard Medical School, Boston, MA USA

**Keywords:** Healthy lifestyle, Metabolomics, Metabolite profile, PREDIMED trial

## Abstract

**Background:**

A healthy lifestyle (HL) has been inversely related to type 2 diabetes (T2D) and cardiovascular disease (CVD). However, few studies have identified a metabolite profile associated with HL. The present study aims to identify a metabolite profile of a HL score and assess its association with the incidence of T2D and CVD in individuals at high cardiovascular risk.

**Methods:**

In a subset of 1833 participants (age 55-80y) of the PREDIMED study, we estimated adherence to a HL using a composite score based on the 2018 Word Cancer Research Fund/American Institute for Cancer Research recommendations. Plasma metabolites were analyzed using LC-MS/MS methods at baseline (discovery sample) and 1-year of follow-up (validation sample). Cross-sectional associations between 385 known metabolites and the HL score were assessed using elastic net regression. A 10-cross-validation procedure was used, and correlation coefficients or AUC were assessed between the identified metabolite profiles and the self-reported HL score. We estimated the associations between the identified metabolite profiles and T2D and CVD using multivariable Cox regression models.

**Results:**

The metabolite profiles that identified HL as a dichotomous or continuous variable included 24 and 58 metabolites, respectively. These are amino acids or derivatives, lipids, and energy intermediates or xenobiotic compounds. After adjustment for potential confounders, baseline metabolite profiles were associated with a lower risk of T2D (hazard ratio [HR] and 95% confidence interval (CI): 0.54, 0.38–0.77 for dichotomous HL, and 0.22, 0.11–0.43 for continuous HL). Similar results were observed with CVD (HR, 95% CI: 0.59, 0.42–0.83 for dichotomous HF and HR, 95%CI: 0.58, 0.31–1.07 for continuous HL). The reduction in the risk of T2D and CVD was maintained or attenuated, respectively, for the 1-year metabolomic profile.

**Conclusions:**

In an elderly population at high risk of CVD, a set of metabolites was selected as potential metabolites associated with the HL pattern predicting the risk of T2D and, to a lesser extent, CVD. These results support previous findings that some of these metabolites are inversely associated with the risk of T2D and CVD.

**Trial registration:**

The PREDIMED trial was registered at ISRCTN (http://www.isrctn.com/, ISRCTN35739639).

**Supplementary Information:**

The online version contains supplementary material available at 10.1186/s12933-023-01912-6.

## Introduction

The role of lifestyle factors in health has been the object of numerous studies in recent decades. Most of the studies report a strong relationship between lifestyle and chronic disease morbidity and mortality [1–3]. Unhealthy lifestyle behaviors including physical inactivity, unhealthy diet, smoking, excess alcohol consumption, and stress have been individually associated with an increased risk of type 2 diabetes (T2D) and cardiovascular disease (CVD) [4–9].

Because of the synergistic effect of lifestyle behaviors, several lifestyle scores have been proposed to assess the risk of chronic diseases. Some of these scores have been validated, demonstrating greater reductions in T2D or CVD risk compared to the expected reduction from the individual lifestyle factors included in the score [10, 11]. One of these scores was operationalized based on the 2018 recommendations of the World Cancer Research Fund/American Institute for Cancer Research (WCRF/AICR) [12]. This score considers lifestyle components such as (1) healthy weight, (2) physical activity, (3) intake of fiber from plant foods, (4) fast food and processed foods consumption, (5) red and processed meat consumption, (6) sugar-sweetened beverage consumption, (7) alcohol intake, (8) supplements for cancer prevention and (9) breastfeeding [13]. Interestingly, unlike other low-risk lifestyle scores, the WCRF/AICR score does not consider the overall diet as a single component and enables to evaluate of the synergy between nutritional components. Higher 2018 WCRF/AICR scores have been prospectively associated with cancer and CVD mortality in older adults [14] and also with the risk of T2D [15].

Various clinical trials have demonstrated that lifestyle interventions can have a beneficial effect on the intermediate metabolism and cardiovascular risk factors, reducing the risk of T2D or CVD incidence [16–18]. With the recent development of omics technologies, the identification of metabolomics signature profiles associated with HL may be of interest to better understand which metabolic pathways are involved in the development of disease and can provide useful information for designing appropriate preventive interventions.

Recently, two studies have identified metabolic signatures reflecting a healthy lifestyle pattern which were inversely associated with the risk of cancer [19, 20]. However, to the best of our knowledge, only one study has been conducted to assess the association of a composite measure of lifestyle with plasma metabolite profiles and incident T2D, and whether these metabolites could explain the prospective association between a Healthy Lifestyle (HL) and incident T2D [16]. In that study, the proposed HL score showed a strong inverse association with T2D, which was largely explained by a set of plasma metabolites measured years before the clinical diagnosis.

Therefore, the aim of the present study was to identify a metabolomic profile of the WCRF/AICR lifestyle score and to relate this metabolomic profile to the risk of T2D and CVD in a subset of participants of the PREvención con DIeta MEDiterránea (PREDIMED) study.

## Methods

### Study population

#### Study design

This study was carried out in the frame of the PREDIMED study, a multicenter randomized controlled trial conducted in Spain from 2003 to 2010 designed to assess the effect of the Mediterranean diet (MedDiet) on the primary prevention of CVD in a population at high cardiovascular risk. Participants were aged between 55 and 80 years and had no CVD at enrollment, but they were at high risk because of the presence of type 2 diabetes or at least three of the following risk factors: current smoking, hypertension, hypercholesterolemia, low high-density lipoprotein (HDL)-cholesterol, overweight or obesity, and family history of premature CVD. Exclusion criteria included any severe chronic illness, drug or alcohol addiction, or allergy or intolerance to olive oil or nuts, two key supplemental foods. In the main study, participants were randomly assigned to three intervention groups: a MedDiet supplemented with virgin olive oil, a MedDiet supplemented with mixed nuts, or a low-fat diet according to the American Heart Association guidelines (control group). A complete description of the PREDIMED study protocol can be found on the study website (http://www.predimed.es/) and in previous publications [21, 22]. The PREDIMED trial was registered at ISRCTN (http://www.isrctn.com/, ISRCTN35739639). All participants in the study provided written informed consent, and the Institutional Review Boards of each of the respective study centers approved the protocol.

#### Discovery and validation population

Participants involved in the discovery population analysis come from three study subsamples designed for metabolite profiling and derived from the PREDIMED study: the PREDIMED-CVD study (primary outcome of the trial) that consisted of 229 incident CVD cases (myocardial infarction, stroke, or death from cardiovascular disease) and 788 sub-cohort participants without CVD at baseline (with an overlap of 37 participants) [23, 24], the PREDIMED-T2D study (secondary outcome) that consisted of 251 incident T2D cases (based on at least one of the following criteria: current treatment with insulin or oral hypoglycemic drugs, fasting glucose > 126 mg/dl or glucose > 200 mg/dl in two measurements after an oral glucose tolerance test (OGTT)) and 694 sub-cohort participants without T2D at baseline (overlapping n = 53) [25, 26], and a third subset of PREDIMED participants who completed an OGTT at baseline (n = 132).

All participants that have baseline metabolomics data available from the aforementioned studies and have completed validated semi-quantitative 137-item food frequency questionnaires (FFQs) were included to identify the HL profile (n = 1882). Participants with data missing from their FFQs at baseline (n = 11), a daily energy intake of < 500 and > 3500 kcal/day for women or < 800 and > 4000 kcal/day for men (n = 34), or ≥ 20% missing metabolite values (n = 4) were excluded from the analysis. The final sample included a total of 1833 participants at baseline (discovery population), of whom 633 were allocated to the MedDiet group supplemented with extra virgin olive oil, 629 to the MedDiet group supplemented with the nuts, and 571 to the control diet group (Supplemental Fig. 1).

To validate the results, an internal validation analysis was conducted in the same population for whom lifestyle and metabolomics data were available after 1 year of follow-up. Participants were excluded if they had missing values in FFQs (n = 269) and lifestyle habits (n = 10), a daily energy intake between < 500 and > 3500 kcal/day for women or < 800 and > 4000 kcal/day for men (n = 22) or ≥ 20% missing metabolite values (n = 57) at 1 year. The final sample at the 1-year visit (validation sample) was 1524 participants (Supplementary Fig. 1).

### Descriptive data and sample collection

At baseline and yearly after, participants provided general descriptive information on sociodemographics (sex, age, level of education, and civil status), lifestyle (smoking habits, physical activity), disease history, and drug use, among other things by answering general questionnaires in a face-to-face interview. Physical activity was assessed using a Spanish-validated version of the Minnesota leisure-time physical activity questionnaire [27, 28]. Additionally, anthropometric variables (weight, height, waist circumference) were measured and blood samples were obtained in fasting conditions by trained nurses. A semi-quantitative validated FFQ with 137 food items was administered at baseline and at 1 year of follow-up by trained dietitians in face-to-face interviews [29]. The frequency of consumption of the food items was reported using an incremental scale with nine categories (never or almost never; 1–3 servings/month; 1 serving/week, 2–4 servings/week, 6 − 5 servings/week, 1 serving/day, 2–3 servings/day, 4–6 servings/day and > 6 servings/day). Energy and nutrient intake were estimated according to Spanish food composition Table [30].

### Adherence to the lifestyle pattern

An 8-point composite HL score was constructed using the 2018 WCFR/AICR recommendations for cancer prevention. It included the following components: (1) healthy weight, (2) physical activity, (3) plant food, (4) fast food and processed food, (5) red and processed meat, (6) sugar-sweetened beverages, and (7) alcohol consumption. Because of the importance of smoking as a risk factor for chronic disease and morbidity, smoking status was included as another component. Healthy smoking status was defined as low for current smokers, intermediate for former smokers, and high for those who had never smoked. For each healthy factor, participants received a score of 1 point for high adherence, 0.5 points for intermediate adherence, or 0 points for low adherence. However, breastfeeding and multivitamin supplement use for cancer prevention were omitted in our analysis due to the lack of information in relation to breastfeeding, and to the extremely low occurrences of the use of supplements for cancer in our population. More detailed information on the cut-off used for each component of the score has been previously reported[13] (see Table S1). The sum of these 8 components provided a total HL score ranging from 0 to 8 (in increments of 0.5 points), where higher scores indicate a healthier lifestyle.

### Metabolomics profiling

For the metabolomics analysis, fasting blood samples were collected at baseline and after 1 year in EDTA-containing tubes and stored in freezers at − 80 °C. Participant samples were then randomly ordered and analyzed in pairs (at baseline and 1-year follow-up visits) for quantitative metabolic profiling. Metabolomics assays of the plasma samples were performed at the Broad Institute using high-throughput two-liquid chromatography-tandem mass spectrometry (LC-MS) [31]. Methods to measure polar and non-polar metabolites have been previously described [32–34]. Briefly, amino acids (AAs) and other polar metabolites were profiled with a Nexera X2 U-HPLC (Shimadzu Corp., Marlborough, MA, USA) coupled to a Q-Exactive mass spectrometer (Thermo Fisher Scientific, Waltham, MA, USA). These metabolites were extracted from 10µL plasma using 90µL of solution with acetonitrile/methanol/formic acid (74.9:24.9:0.2 v/v/v) with stable isotope-labeled internal standards: valine-d8 (Sigma-Aldrich) and phenylalanine-d8 (Cambridge Isotope Laboratories). The samples were centrifuged for 10 min at 9000 × g at 4 °C. The supernatants were injected directly into a 150 × 2-mm, 3-µm Atlantis HILIC column (Waters), and eluted isocratically at a flow rate of 250µL min^− 1^ with 5% of 10 mmol ammonium formate L^− 1^ and 0.1% formic acid in water (mobile phase A) for 0.5 min followed by a linear gradient to 40% of acetonitrile with 0.1% formic acid (mobile phase B) for over 10 min. Fatty acids and other lipids were profiled using a Nexera X2 U-HPLC (Shimadzu Corp., Marlborough, MA, USA) coupled with an Exactive Plus Orbitrap MS (Thermo Fisher Scientific, Waltham, MA, USA). These aliphatic metabolites were extracted from 10µL plasma using 190 µL of isopropanol containing 1,2-didodecanoyl-sn-glycerol-3-phosphocholine (Avanti Polar Lipids) as an internal standard. The lipid extraction (2µL) was injected into a 100 × 2.1 mm, 1.7 μm ACQUITY BEH C8 column (Waters; Milford, MA). The column was eluted isocratically with 80% mobile phase A: 10 mM ammonium acetate/methanol/formic acid (95:5:0.1 v/v/v) in 1 min followed by a linear gradient to 80% of mobile phase B: methanol/formic acid (99.9:0.1 v/v) in 2 min, then for a linear gradient to 100% mobile-phase B in 7 min, and finally 3 min of 100% of mobile-phase B.

Mass spectrometry analyses were carried out using electrospray ionization in the positive-ion mode, and full-scan spectra were acquired over 70–800 *m/z* for AAs and over 200–1100 *m/*z for lipids. Raw data were processed using Trace Finder 3.1 and 3.3 (Thermo Fisher Scientific) and Progenesis QI (Nonlinear Dynamics). Polar metabolite identities were confirmed using authentic reference standards and lipid metabolites were identified using the polar head group, total acyl carbon numbers, and total acyl double bond content. Pooled plasma reference samples were analyzed, in pairs, at intervals of 20 participant samples to standardize the data and assess the quality across the sample batches and analytical queue. From each pooled reference pair, one sample functioned as a passive quality control (QC) to assess the analytical reproducibility of each metabolite measurement, while the other one was used to standardize the process with a “nearest neighbor” approach.

Standardized values were generated using the ratio of the value in each sample over the nearest pooled plasma reference and multiplied by the median value measured across the pooled references. After quality filtering and standardization, 399 named metabolites qualified for primary analyses. Three metabolites (1,2-didodecanoyl-sn-glycero-3-phosphocholine, valine-d8, and phenylalanine-d8) were removed because they were internal standards, and 11 more because they had a high number of missing values (i.e., > 20%). In total, 385 metabolites were used in the final analysis.

### Statistical analysis

Descriptive data of the study participants are presented using percentages and counts for categorical variables or mean and standard deviations (SD) for quantitative traits. To identify a metabolic signature associated with the HL, a continuous HL score was associated with the final dataset of 385 metabolites (inverse-normal transformed) from the PREDIMED study at baseline used as the discovery population with a cross-validation approach. Then, data from the 1-year follow-up was used as a self-validation sample. Values missing from selected metabolites (with more than 80% of the data available) were imputed using the random forest imputation approach (“missForest” function from the “missForest” R package) as previous publications have recommended [35–37]. Due to the high dimensionality and collinear nature of the data, logistical (binomial) linear regression models with the elastic net penalty, to reduce model overfitting, were used to build a predictive model for HL classification based on metabolic data (“glmnet” R package). The elastic net algorithm is controlled by parameter α, the parameter α was trained in 0.1 increments from 0 (Ridge regression) to 1 (Lasso regression). To train the method, a leave-group-out cross-validation approach was performed with 10 subsets of the training data in the discovery population. Elastic net regression was applied to all but one subset, and the estimated model was applied to the left-out subset. The best prediction accuracy was obtained with 𝛼=0.1. Furthermore, an additional 10-fold cross-validation (CV) was performed to determine the optimal tuning of the 𝜆 parameter, which corresponds to the minimum mean squared error (minMSE). To reduce the number of retained predictors, metabolites were estimated with the minMSE + 1SE parameter using the argument s= “lambda.1se” in the cv.glmnet function of the “glmnet” R Package. For reproducibility purposes, a 10-CV elastic net regression with minMSE + 1SE parameter was replicated 10 times in all participants and the average value of the regression coefficients was reported. To evaluate the reliability of the results, the metabolite signature of the HL score was validated by Pearson correlation coefficients.

Cox’s proportional hazards regression with Barlow weights and a robust variance estimator was used to associating HL and the metabolic signature with incident T2D and CVD risk. For each T2D and CVD nested case-cohort studies, three Cox models with different covariates were estimated with 245 events and 222 events, respectively, at baseline. The first model was adjusted for age, sex, and propensity scores (see Supplementary methods) [22, 38] and stratified by recruiting center and intervention group. The second model was further adjusted for education level (primary, secondary, or college), family history of premature coronary heart disease (CHD) (yes/no), hypercholesterolemia (yes/no), cholesterol-lowering medications (yes/no), hypertension (yes/no), antihypertensive treatment (yes/no) and total energy intake (kcal per day). Diabetes prevalence (yes/no) was included as a covariate in the Cox models using the CVD sub-cohort. In the third model, the self-reported HL categorical variable was included to examine association independence. For the 1-year analyses (i.e., validation sample), the same metabolomics models were generated using the 1-year follow-up metabolomics data, excluding incident cases of T2D or CVD that occurred before the 1-year follow-up visit for the case-cohort studies (161 and 159 events, respectively).

Several sensibility analyses were done. Participants were classified into two categories of HL score (low, < 4 points or high, ≥ 4 points) at baseline and 1-year follow-up visits, and elastic net regression models were done again (𝛼=0.6). The performance of the predictive model for the HL categorical variable was evaluated by the area under the curve (AUC) of the receiver operating characteristic (ROC) generated. The sample was stratified by intervention groups designed on the PREDIMED trial (MedDiet supplemented with extra virgin olive oil, MedDiet supplemented with nuts, and control group). Associations between the high or low HL scores and the risk of developing T2D and CVD were calculated in the three intervention groups using Cox regression models with Barlow weights, and the likelihood ratio test was used to assess the significance of the 1-df interaction product-term (effect modification in multiplicative scale) between the intervention groups (MedDiet groups compared with control) and the HL metabolite profile as a categorical variable.

Statistical procedures were performed with Stata 14.2 software for Windows (Stata Corp.) and R software v4.2.1 (www.R-project.org) (R Core Team, 2021). A *p*-value less than 0.05 was considered statistically significant.

## Results

1. Characteristics of the study participants.

A total of 1883 participants (58% women, 67 years) at baseline and 1524 participants (57% women, 67 years) at 1-year follow-up, were considered for the present study. The flow chart of selected participants can be seen in Supplemental Figure S1. Table 1 summarizes the characteristics of the study participants in each of the HL categories. The mean and SD value of the HL score for participants with low HL scores was 3.01 ± 0.52 at baseline, and 3.05 ± 0.52 after 1 year of follow-up. Those participants in the high HL category showed a score of 4.46 ± 0.51 at baseline and a score of 4.56 ± 0.58 after 1 year. Participants with high HL scores were more likely to be women and older than participants with low HL scores, who had a higher prevalence of T2D, hypercholesterolemia, hypertension, and a positive family history of CVD. Similar trends for these variables were observed at the 1-year visit. By intervention group, the baseline mean HL was 3.67 ± 0.89 for participants in the MedDiet + EVOO group, 3.76 ± 0.90 for participants in the MetDiet + Nuts group, and 3.70 ± 0.88 for participants in the control group. No differences were observed between groups (*P-*value = 0.153).

**Table 1:** Baseline demographic characteristics of study participants according to Healthy Lifestyle (HL; low and high) categories

	Discovery sample		Validation sample	
	Low HL (n = 943)	High HL (n = 890)	Low HL (n = 660)	High HL (n = 864)
HL score*	3.01 ± 0.52	4.46 ± 0.51	3.21 ± 0.76	4.13 ± 0.76
Age (years)	66.42 ± 6.12	68.02 ± 5.89	66.63 ± 5.97	67.48 ± 5.95
Sex (women)	45.39 (428)	70.45 (627)	45.85 (296)	66.78 (577)
BMI (kg/m^2^)	30.76 ± 3.52	29.01 ± 3.38	30.61 ± 3.40	29.18 ± 3.48
Waist circumference (cm)	103.20 ± 9.38	97.26 ± 9.87	103.08 ± 9.55	97.82 ± 10.01
Fasting plasma glucose (mg/dl)	114.27 ± 34.91	111.94 ± 36.77	114.10 ± 36.82	111.78 ± 34.73
T2D % (n)	28.31 (267)	30.00 (267)	30.76 (203)	29.98 (259)
Prediabetes % (n)	24.60 (232)	20.56 (183)	21.82 (144)	22.92 (198)
Hypercholesterolemia % (n)	74.55 (703)	79.21 (705)	71.21 (470)	78.59 (679)
Hypertension % (n)	85.59 (808)	89.03 (795)	85.94 (886)	87.85 (477)
Family history of CVD % (n)	22.14 (209)	27.10 (242)	23.67 (149)	27.81 (233)
Smoking habit % (n)				
Never smoked	41.99 (396)	78.20 (696)	39.79 (256)	74.88 (647)
Used to smoke	31.28 (295)	17.87 (159)	33.03 (218)	19.10 (165)
Currently smoke	26.72 (252)	3.93 (35)	28.18 (186)	6.02 (52)
MVPA (min/week)	12.97 ± 28.89	29.76 ± 42.11	18.47 ± 33.67	24.75 ± 39.79
Fiber (g/day)	23.54 ± 7.56	27.09 ± 9.12	24.41 ± 8.12	26.56 ± 9.22
Fast food & processed food (servings/day)	9.31 ± 5.66	8.04 ± 6.19	9.80 ± 6.43	8.16 ± 6.11
Red meat (g/week)	59.99 ± 39.25	41.08 ± 32.69	57.27 ± 37.28	46.84 ± 34.45
Processed meat (g/day)	29.10 ± 17.99	22.05 ± 15.52	28.82 ± 18.43	23.79 ± 16.77
Sugar-sweetened beverages (g/day)	28.56 ± 76.78	8.55 ± 35.78	24.06 ± 72.55	14.69 ± 50.68
Alcohol consumption (g/day)				
in women	5.60 ± 7.85	2.47 ± 5.59	5.87 ± 8.39	2.91 ± 5.88
in men	19.90 ± 21.41	12.46 ± 15.87	20.75 ± 21.59	12.85 ± 14.84

Descriptive data in both discovery and validation samples were expressed as a percentage (n) for categorical variables and mean ± SD for quantitative variables. Participants were classified in the low group when HL scores were < 4 points or in the high group when ≥ 4 points.

BMI, Body Mass Index; MVPA, Moderate to Vigorous Physical Activity; T2D, Type 2 Diabetes; CVD, Cardiovascular Disease. Prediabetes status was defined as having fasting plasma glucose between ≥ 100 mg/dl and ≤ 125 mg/dl in the absence of the use of drugs for diabetes control.

*The Healthy Lifestyle score was calculated based on 8 factors: (1) a healthy weight, (2) physical activity, (3) plant foods, (4) fast food and processed foods, (5) red and processed meat, (6) sugar-sweetened beverages, (7) alcohol, and (8) smoking. Higher scores indicated better adherence to healthy lifestyle recommendations

2. Identification of metabolites related to a Healthy Lifestyle.

**Fig. 1:**
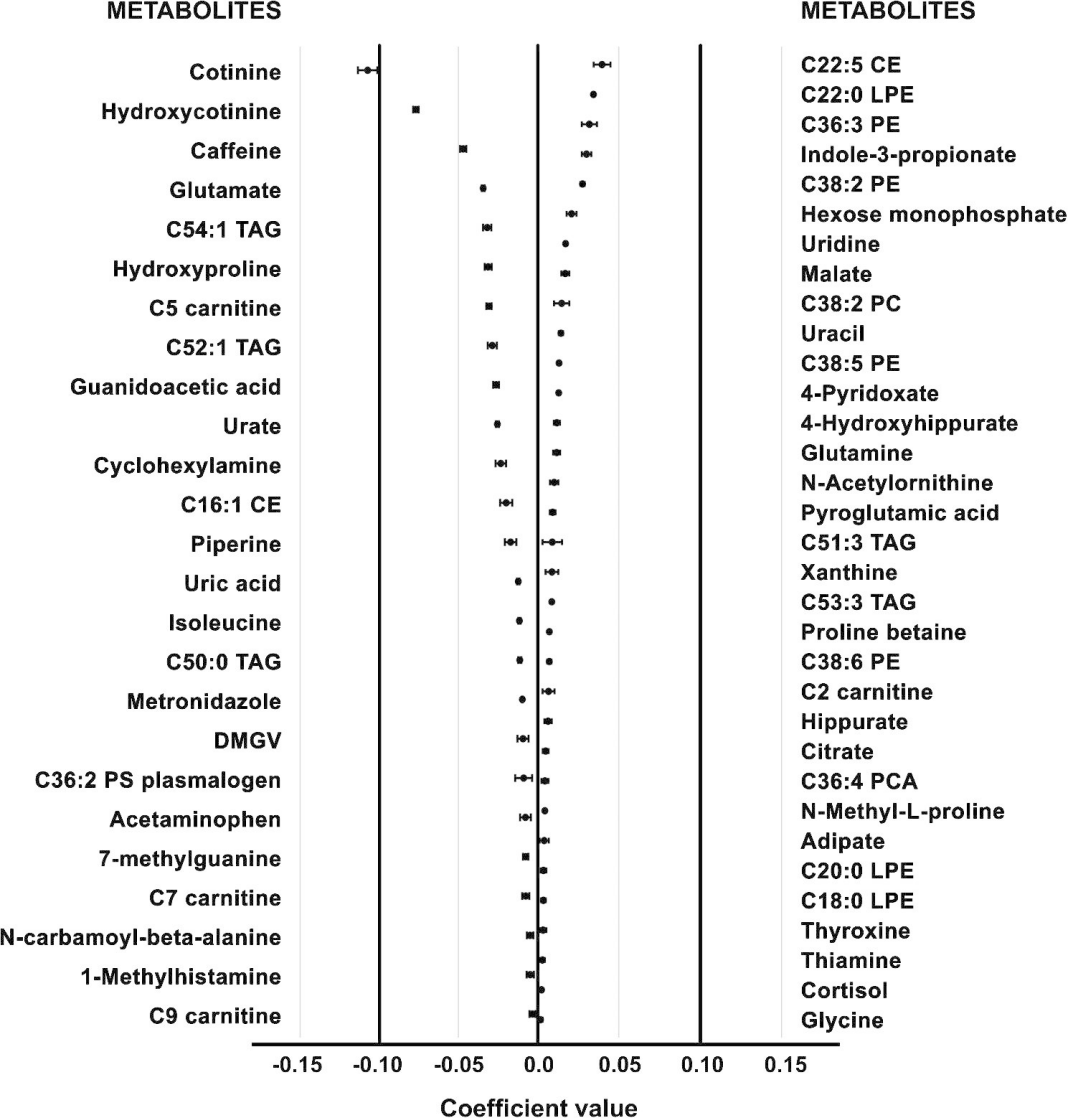
Regression coefficients (mean and SD) of the 58 metabolites selected using the HL score. Metabolites were selected ten times in the 10-cross-validation elastic net regression in the whole dataset (n = 1833). Metabolites with negative coefficients (n = 25) are plotted on the left-hand side, whereas those with positive coefficients (n = 33) are plotted on the right-hand side

The metabolite profile of the HL score (using this score as a continuous variable) obtained by the elastic regression identified 58 metabolites (Fig. 1). Additionally, the low and high HL scores identified 24 metabolites (Figure S3). Table 2 summarizes the predictive performance of the metabolic signature on the HL score at baseline (discovery population) and at 1 year of follow-up (validation population). The Pearson correlation values between the identified metabolite profile and the HL score were 0.50 (95% CI: 0.47, 0.54) in the discovery sample, and 0.44 (95% CI: 0.40, 0.48) in the validation sample (Table 2). The correlation between the HL score and the values predicted by the regression model is represented in Figure S2 as a scatterplot. The mean values of the metabolites’ regression coefficients are reported in Table S2. Regarding sensibility analysis, the AUC values of the ROC analysis were 0.73 (95% CI: 0.70, 0.75) and 0.68 (95% CI: 0.65, 0.71) at baseline and the 1-year visit, respectively.

**Table 2:** Performance of the HL predicted values using the metabolite profile identified and the HL score as the categorical or continuous variable at baseline (discovery) and at 1 year of follow-up (validation)

	Baseline visit (n = 1833)				1-year visit (n = 1524)
Assessment	AUC or correlation coefficient (95% CI)	Total metabolites^c^	Metabolites with positive coefficients	Metabolites with negative coefficients	AUC or correlation coefficient (95% CI)
HL categories	0.73 (0.70, 0.75)^a^	24	13	11	0.68 (0.65, 0.71) ^a^
HL score	0.50 (0.47, 0.54) ^b^	58	25	33	0.44 (0.40, 0.48) ^b^

^a^AUC of the ROC derived from the HL predicted values using the metabolite profile

^b^The Pearson’s coefficients derived from the correlation between the HL score and the predicted values using the metabolite signature within the discovery and validation dataset

^c^Number of metabolites obtained after the ten iterations of the 10-fold cross-validation procedure for the elastic net regression

AUC, area under the curve; ROC, receiver operating characteristic; HL, Healthy Lifestyle

**Fig. 2:**
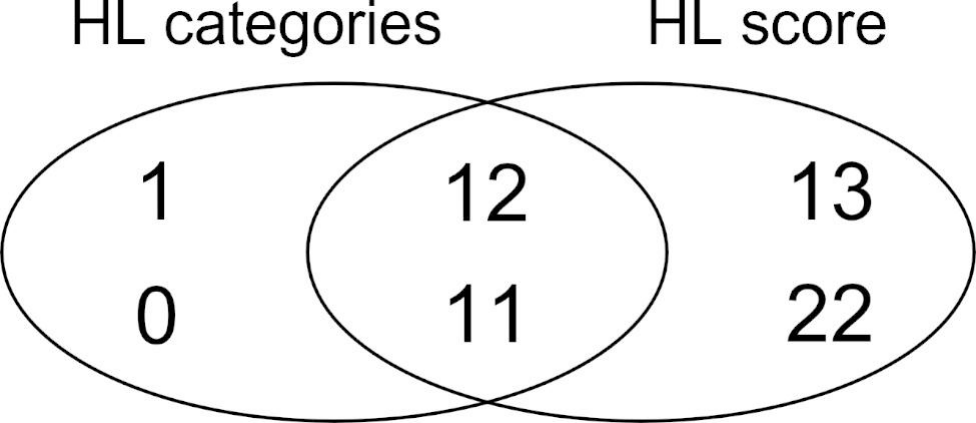
Venn diagram showing the number of metabolites identified using the HL score as the categorical or continuous variable Metabolites were selected with the elastic net regression model in the whole dataset (n = 1833). The number of metabolites with negative and positive coefficients was reported at the top or bottom of the figure, respectively. The metabolites selected with both models appear in the overlapping area of the figure

A total of 23 metabolites (12 negatively associated and 11 positively associated) were selected in both models using the HL as a categorical or continuous variable. The differences and similarities in the metabolite profiles identified using the two models are shown in Fig. 2. The metabolites with negative regression coefficients in both models were cotinine, hydroxycotinine, caffeine, urate, c5 and c7 carnitine, 16:1 cholesterol ester (CE), hydroxyproline, C54:1, C52:1 triacylglycerol (TAG), glutamate and isoleucine. The metabolites with positive coefficients were C22:5 CE, indole3propionate, C36:3 and C38:2 phosphatidylethanolamine (PE), histidine, hexosemonophosphate, C22:0 lysophosphatidylethanolamine (LPE), pyridoxate, C53:3 TAG, cortisol, hydroxyhippurate, and n-methylproline.

3. Association between the identified metabolite profiles and the incidence of T2D and CVD.

Multivariable weighted Cox regression analyses showed a significant inverse association between the baseline HL metabolic signature (explored as a categorical or continuous variable) and the risk of T2D incidence. These results were maintained after adjustment for the self-reported HL score (Table 3). Similar significant associations were found between the 1-year metabolite signature and the incidence of T2D when the incident cases during the first year of follow-up were excluded (Table 3).

**Table 3:** Hazard ratio (95% CIs) for incident T2D and CVD using the metabolomic signatures of the HL score as categorical or continuous variables in the T2D and CVD nested case-cohort studies

	Baseline visit					1-year visit			
	HL categories^a^		HL score^b^			HL categories^a^		HL score^b^	
	HR (95% CI)	*P*	HR (95% CI)	*P*		HR (95% CI)	*P*	HR (95% CI)	*P*
**Type 2 diabetes**									
Incident cases/total participants	245/923					161/705			
Model 1	0.52 (0.38, 0.71)	< 0.001	0.21 (0.12, 0.39)	< 0.001		0.57 (0.36, 0.91)	0.018	0.22 (0.08, 0.55)	0.001
Model 2*	0.51 (0.37, 0.71)	< 0.001	0.20 (0.11, 0.37)	< 0.001		0.46 (0.28, 0.78)	0.004	0.14 (0.05, 0.39)	< 0.001
Model 3	0.54 (0.38, 0.77)	0.001	0.22 (0.11, 0.43)	< 0.001		0.56 (0.32, 0.95)	0.032	0.18 (0.06, 0.58)	0.004
**Cardiovascular diseases**									
Incident cases/total participants	222/980					159/916			
Model 1	0.64 (0.47, 0.87)	0.005	0.38 (0.22, 0.67)	0.001		0.76 (0.54, 1.07)	0.116	0.54 (0.29, 0.99)	0.045
Model 2*	0.64 (0.47, 0.88)	0.006	0.42 (0.24, 0.72)	0.002		0.72 (0.48, 1.06)	0.099	0.52 (0.27, 1.01)	0.055
Model 3	0.59 (0.42, 0.83)	0.002	0.58 (0.31, 1.07)	0.080		0.82 (0.55, 1.22)	0.339	0.87 (0.43, 1.77)	0.703

Cox proportional hazard models with Barlow weights were used to estimate the HRs for T2D and CVD risk

^a^ HRs between HL categories using low HL as reference

^b^ HRs refers to a 1-point increment in the metabolite profile of the HL score

Model 1: metabolite profile adjusted for age, sex, and propensity scores and stratified by recruitment center and intervention group. Model 2: model 1 + education level, family history of CHD, hypercholesterolemia, cholesterol-lowering medications, hypertension, antihypertensive treatment, and total energy intake

Model 3: model 2 + self-reported HL score (as categorical or continuous)

*Model 2 used in the CVD case cohort was further adjusted for diabetes prevalence

HL, healthy lifestyle; HR, hazard ratio; CI, confidence interval; P, p-value

In the case of the CVD-case cohort, a significant inverse association was observed between the HL metabolic signature of the HL at baseline (explored as a categorical variable) and the risk of CVD incidence (Table 3). However, this inverse association was attenuated when the HL score was evaluated as a continuous variable. In the adjusted models, no associations were found between the 1-year metabolite signature and the incidence of CVD after the incident cases that occurred during the first year of follow-up had been excluded (Table 3).

The *P* for interaction between the control group and the MedDiet + EVOO group was only significant for T2D (*P* = 0.017 for T2D and *P* = 0.203 for CVD), and non-significant in the case of the MedDiet + Nuts group (*P* = 0.088 for T2D and *P* = 0.755 for CVD). We explored the associations splitting the population by the intervention group, and similar inverse associations were found (Table S3).

## Discussion

In this study, a metabolic signature of a predefined HL score was identified in the PREDIMED population using an agnostic machine-learning approach. The metabolic signature was inversely associated with the incidence of diabetes independently of the HL score and to a lesser extent with the risk of CVD. To the best of our knowledge, this is the first study to identify plasma metabolite signatures of a lifestyle score that predict the risk of future T2D and CVD in individuals at high cardiovascular risk, using data at baseline and after 1 year of follow-up.

In the last decade, some of the metabolite profiles identified with dietary patterns [39–41], lifestyle behaviors [42, 43], and lifestyle scores [16] have been related to the risk of various chronic conditions [19, 20]. For example, in PREDIMED, a metabolic signature that robustly reflects adherence and metabolic response to a Mediterranean diet prospectively predicts the risk of CVD risk independently of traditional risk factors in both Spanish and US cohorts [18]. In the Coronary Artery Risk Development in Young Adults (CARDIA) study, dietary metabolic signatures have also been associated with long-term diabetes and cardiovascular risk [44]. Metabolic signatures of physical activity have also been recently reported [42].

However, few studies have analyzed the associations between the metabolomic signatures of healthy lifestyle scores and the risk of future disease. Two studies identified a metabolic signature reflecting a HL pattern that was inversely associated with cancer risk [19, 20]. Unfortunately, only in a few studies have plasma metabolite profiles of HL scores been explored and prospectively related to the risk of cardiometabolic conditions [17, 45].

To the best of our knowledge, only one study has been conducted to identify the plasma metabolite signature of a composite measure of lifestyle, and whether these metabolites can prospectively explain the association between a HL and incident T2D [16]. The HL score used in that study showed a strong inverse association with T2D incidence, which was largely explained by a set of plasma metabolites measured years before the clinical diagnosis. In the case of CVD, data from the UK biobank was recently used to associate the metabolic signature of a validated HL score with the incidence of coronary artery disease that improved the prediction of coronary artery disease risk using classical cardiovascular risk factors [45].

Our study identified two metabolic signatures of the HL score (categorical or continuous) with 24 and 58 selected metabolites. The performance analysis confirms that the correlation between self-reported and predicted HL scores is quite strong. Selected metabolites may be used as potential biomarkers of lifestyle and might provide a better understanding of the mechanism underlying the benefits of HL. However, this requires confirmation in other studies and populations. Most of the overlapping selected metabolites for both signatures (12 of which were negatively associated with HL and 11 positively) were amino acids and derivatives, lipids, metabolites involved in the intermediate energy metabolism, xenobiotics, and products of bacterial co-metabolism.

Most of the metabolites identified had been previously reported to be highly related to the lifestyle factors included in the 2018 WCRF/AICR HL score we used to build our metabolite signatures (healthy weight, physical activity, fiber from plant foods, fast food, and processed foods, red and processed meat, sugar-sweetened beverages, and alcohol consumption) [13].

Concerning the healthy body weight component of the HL score, our results are in line with previous studies that reported associations between some amino acids, lipids, and their derivative metabolites with body weight status, BMI, and fat mass [46–48]. Branched-chain amino acids (BCAAs), such as leucine, isoleucine, and valine, are the most common ones to be associated with obesity, while only glycine has been inversely associated with increased fat mass [46, 48]. Furthermore, higher BCAA levels and lower glycine and glutamine concentrations have been linked to insulin resistance and a higher risk of T2D [49]. Likewise, elevated glutamate concentrations have been related to higher BMI and insulin resistance [49, 50]. Lipid metabolites, such as short- and long-chain acylcarnitines, fatty acids (particularly pro-inflammatory fatty acids), and phospholipids, have been related to adiposity, increased body weight, insulin resistance, and glucose metabolism [48, 49]. In our study, isoleucine, glutamate, and some lipid metabolites (C5 and C7 carnitine, C52:1 and C54:1 TAG) were negatively associated with the HL score, while glycine, PE (C36:3 and C38:2), and LPC (C18:0 and C22:0) were positively associated. The observed associations might reflect the healthy body weight score component and may contribute to explaining the decreased risk of CVD and T2D observed for the HL metabolite signature, as some of these metabolites have been previously associated with an elevated risk of T2D and CVD [49].

As far as physical activity is concerned, in 7271 men from the Finnish cohort METabolic Syndrome In MEN (METSIM), increased physical activity was significantly associated with high levels of choline plasmalogens, lysophosphatidyl cholines, polyunsaturated fatty acids, long-chain acylcarnitines, imidazoles, bilirubin, hydroxy acids, indole propionate, and indole lactate [42]. Several of these metabolites have been previously associated with a decreased risk of T2D or CVD and a healthy diet. Conversely, individuals with increased physical activity showed lower levels of diacylglycerols, monoglycerols, phosphatidylcholines, phosphatidylethanolamines, phosphatidyl inositol, sphingolipids, bile acids, steroids, short-chain acyl carnitines, γ-glutamyl-amino acids, N-acyl-L-α-amino acids, glutamate, creatine, tyrosine, pyruvate, and lactate than physically inactive individuals [42]. As in the METSIM study, in our study indole propionate was also directly associated with the HL, while short-chain acylcarnitines (C5 C7, and C9) and glutamate were inversely associated, which probably reflects the physical activity component of the score. Interestingly, indole propionate and glutamate have been, negatively and positively, related, respectively to an increased risk of diabetes [49], as was also observed with our HL metabolomic signature. However, the levels of acylcarnitine C5 were unexpectedly negatively associated with the HL score when this metabolite has been associated with an increased risk of diabetes [49].

Some metabolites related to HL in our study were previously associated with a plant-based diet [51, 52]. In a general, healthy population from the USA, a plant-based diet was inversely linked with isoleucine, hydroxyproline, C5 carnitine, two plasmalogen subclasses, and three triacylglycerols (C51:0, C:48:0, and C52:0) [52]. Meanwhile, direct associations were described for trigonelline, hippurate, betaine, pipecolic acid, pantothenic acid, N-acetyl ornithine, C22:0 LPE, and C58:11 TAG [52]. Further, the metabolic signature of the healthy plant-based diet was associated with a 15% lower risk of T2D [53]. In this regard, the role of isoleucine, hydroxyproline, and C5 carnitine in T2D and CVD risk has been well established [49, 53], whereas hydroxy hippurate and indol-3-propionate gut microbiota metabolites have proved to be decreased in individuals with T2D [54, 55]. In our study, isoleucine, hydroxyproline, C5 carnitine, hippurate, hydroxy hippurate, and indol-3-propionate were observed to have similar associations with the HL score. In the MASALA cohort [51], a “prudent” dietary pattern (high in fruits, vegetables, nuts, and legumes) was associated with proline betaine, LPC (22:4/0:0), LPE (22:4/0:0), PC (18:0/22:4) and SM(d19:1/16:0), but in our analyses, only the association with proline betaine was detected. We also observed a positive relationship between the HL score and n-methyl proline, which has been associated with greater adherence to healthy dietary patterns [41].

Red meat (RM) and processed meat (PM) are considered in the HL score that we used, and consistent evidence has been reported that high consumption of these food products is associated with an increased risk of diabetes and CVD [56]. In a previous analysis conducted in the same PREDIMED study population, consumption of RM and PRM was associated with lower levels of lactate, some carnitines, glycine, C34:0 phosphatidyl ethanolamine (PE), C40:10 PC, C22:1 SM, and uridine, and higher levels of C38:4 PC plasmalogen, isoleucine, leucine, uric acid, and C36:5 PC plasmalogen, cotinine, and cortisol [57]. In the present study, glycine and uridine were positively associated with the HL score, while isoleucine, uric acid, cotinine, and cortisol were negatively associated, which may reflect the lower consumption of RM and PM. Most of the aforementioned metabolites associated with the HL score in our study have been related in the expected direction to the risk of diabetes or CVD in previous studies [49, 58]. As far as cortisol is concerned, it can be considered a core hormonal mediator of the allostatic load produced in response to various stresses. Alterations in morning serum cortisol and daily diurnal cortisol have been associated with adiposity, dyslipidemia, incident diabetes, and CVDs [59].

A higher intake of sugar-sweetened beverages has been prospectively associated with increased levels of dimethyl guanidino valerate (DMGV) in plasma [60]. DMGV has been reported to be a biomarker of liver fat and a predictor of diabetes [34]. It should be pointed out that in our study this metabolite was negatively associated with the HL score. On the other hand, some studies have reported that a high-fructose diet increases de novo purine biosynthesis in humans, which increases the production of uric acid. For example, the intake of high-fructose corn syrup-sweetened beverages induced a dose-dependent increase in circulating lipid/lipoprotein risk factors for CVD and uric acid [61]. Carbonated drinks and fruit juice have also been positively associated with plasma leucine and isoleucine levels, and negatively with aconitic acid and methylmalonic acid [62]. In our study, uric acid and isoleucine, which have both been related to an increased risk of diabetes and CVD [49, 63] were negatively associated with the HL score.

Smoking, one of the most widely recognized unhealthy behaviors, has been associated with an elevated risk of T2D and CVD [4]. In our study, the metabolites of tobacco cotinine and hydroxicotinine were negatively related to low HL, as expected. It has been reported that smokers frequently consume higher amounts of alcohol, meat, and processed food, and have lower levels of physical activity [64], a combination of unhealthy habits that increases the risk of T2D and CVD [1, 16]. In addition, previous studies conducted in the same PREDIMED population, but also in other cohorts, reported that smokers are also more frequent consumers of coffee [65–67]. The inverse association observed between caffeine intake and the HL score in our study may be a reflection of this.

The loss in the significance of the association between the metabolomic HL signature and CVD incidence at 1-year may be explained by the changes in HL that occurred as a consequence of the intervention as well as a reduced number of participants included in this analysis, in spite of the trend of the associations was in the same direction that in the baseline analysis. Additionally, although the direction of the associations between the metabolomics HL signature and the incidence of T2D was similar between the three arms of the trial, the significance was only observed in both MedDiet groups probably because of the effect of the interventions decreasing the risk of diabetes as was previously reported in PREDIMED [68].

Our study has some limitations. First, data obtained by questionnaires may be susceptible to participant recall bias despite being recorded in face-to-face interviews by trained personnel. Measurement errors must be considered a possibility even though the questionnaires were validated in a population similar to PREDIMED [29]. Second, because of insufficient data available, two WCRF/AICR recommendations (breastfeeding and multivitamin supplement use for cancer prevention) were omitted from our HL score. Nonetheless, it is important to mention that these components would have had very little impact on our results since they are only applicable to specific subpopulations. We also must mention that information about renal function was available only in a low percentage of participants. Then, renal function has not been included in the manuscript as a potential confounder in our analysis. Third, participants were selected from an elderly Mediterranean population at high risk of CVD, and the pathogenic factors related to CVD development might influence the results. In addition, because the participants for this study were selected in the context of a case-cohort study design, the number of participants that have developed CVD during the follow-up is higher than in the general population. For this reason, the generalizability of the findings to other populations may be limited and the results should be replicated and validated in other cohorts. Forth, the metabolic signature was estimated with more than 350 well-characterized metabolites, but other unknown relevant metabolites related to HL may exist. Finally, because of its observational nature, our study was not designed to establish an unequivocal cause-effect relationship between identified metabolic signatures of HL and T2D or CVD incidence.

There are also strengths to our study, smoking habit was included in our HL score because of its importance as a risk factor for T2D and CVD. Furthermore, our analyses were conducted in a large cohort and possible confounding was controlled by several covariates. Finally, the results on the association between the metabolite signatures and the risk of T2D and CVD are still in the same direction when analyzed separately by intervention group. This adds robustness to our findings.

In conclusion, a set of metabolites were selected as potential biomarkers of a HL pattern in an elderly Mediterranean population at high risk of CVD. Most of these metabolites are amino acids and derivatives, lipids, xenobiotics, and products of bacterial co-metabolism. This HL metabolomic signature was inversely associated with the risk of incident T2D and to a lesser extent with incident CVD.

### Electronic supplementary material

Below is the link to the electronic supplementary material.

Supplementary Material 1

## Data Availability

The dataset generated and/or analyzed during the current study is not publicly available due to the lack of authorization from PREDIMED participants. Those wishing to access the PREDIMED trial data used in this study can request the corresponding author and it will then be passed on to members of the PREDIMED Steering Committee for deliberation.

## Abbreviations

AA: Amino acid
AICR: American Institute for Cancer Research
AUC: Area under the curve
BCAA: Branched-chain amino acid
BMI: Body mass index
CARDIA: Coronary Artery Risk Development in Young Adults
CE: Cholesterol ester
CV: Cross-validation
CVD: Cardiovascular disease
DAG: Diacyl glycerols
DMGV: Dimethyl guanidino valerate
EDTA: Ethylenediaminetetraacetic acid
FFQ: Food frequency questionnaires
HL: Healthy lifestyle
LC-MS: Liquid chromatography-tandem mass spectrometry
LPC: Lysophosphatidyl choline
LPE: Lysophosphatidyl ethanolamine
MedDiet: Mediterranean diet
METSIM: METabolic Syndrome In MEN
minMSE: Minimum mean squared error
PREDIMED: PREvención con DIeta MEDiterránea
QC: Quality Control
PC: Phosphatidyl choline
PE: Phosphatidyl ethanolamine
PI: Phosphatidyl inositol
PM: Processed meat
RM: Red meat
ROC: Receiver operating characteristics
SD: Standard deviation
SE: Squared error
SM: Sphingomyelin
TAG: Triacylglycerol
T2D: Type 2 diabetes
U-HPLC: Ultra High-Performance Liquid Chromatography
WCRF: Word Cancer Research Fund
