# Supplementary material for: Plasma metabolite profiles associated with the World Cancer Research Fund/American Institute for Cancer Research lifestyle score and future risk of cardiovascular disease and type 2 diabetes
Source: Cardiovasc Diabetol. 2023 Sep 16;22:252. doi: 10.1186/s12933-023-01912-6 (PMC10505328; doi:10.1186/s12933-023-01912-6)
Supplement: Supplementary file 1 — Supplementary Material 1 [file 12933_2023_1912_MOESM1_ESM.docx]

**Supplemental files**

1. Supplementary methods

1.1. Propensity score estimations

Propensity scores are the probability of participants being assigned to one of the PREDIMED intervention groups (i.e., control group with low-fat diet, Mediterranean diet supplemented with extra virgin olive oil, or Mediterranean diet with walnuts). Propensity scores were estimated using a multinomial logistic model with the 3 groups mentioned above (trial assignment) as the outcome. The following co-variables were the predictors of assignment: ethnicity; marital/living alone status; unemployment/retirement/housewife as the only occupation status; presence of any disability; years of education; dyspnea; history of non-atherosclerotic cardiovascular disease, history of kidney disease, chronic lung disease, depression, cataracts, obstructive sleep apnea, cancer; use of vitamin/mineral supplements, angiotensin-converting enzyme inhibitors, diuretics or other antihypertensive medication, statins or other lipid-lowering medication, insulin or oral antidiabetic agents, aspirin/antiplatelet therapy; the score of psychological tension, fasting plasma glucose, ratio of blood total cholesterol to HDL cholesterol, blood LDL-cholesterol levels, and blood triglycerides. The predicted probabilities of being assigned to the Mediterranean diet supplemented with extra virgin olive oil group (P1), or to the Mediterranean diet with nuts group (P2) were maintained. Estimated propensity scores were used as covariates in the Cox regression models to adjust the allocation of each participant in the intervention groups.

Table S1. Defining scoring thresholds for components of the HL score

| HL recommendations | Operationalization of recommendations | Scoring |
| --- | --- | --- |
| 1. Healthy weight | 18.5 ≤ BMI ≤ 24.9 kg/m^2^ | 1 |
|  | 24.9 < BMI < 30 kg/m^2^ | 0.5 |
|  | BMI < 18.5 or BMI ≥ 30 kg/m^2^ | 0 |
| 2. Physical activity | MVPA ≥ 150 minutes/week | 1 |
|  | MVPA ≥ 75 and <150 minutes/week | 0.5 |
|  | MVPA < 75 minutes/week | 0 |
| 3. Fiber from foods | ≥ 30 g/day | 1 |
|  | ≥ 15 and < 30 g/day | 0.5 |
|  | < 15 g/day | 0 |
| 4. Fast food and processed foods | < 1.5 servings/day | 1 |
|  | ≥ 1.5 and < 3 servings/day | 0.5 |
|  | ≥ 3 servings/day | 0 |
| 5. Red and processed meat | RM < 450 g/week and PM < 3 g/day | 1 |
|  | RM < 450 g/week and PM ≥ 3 and < 50 g/day | 0.5 |
|  | RM ≥ 450 g/week and PM ≥ 50 g/day | 0 |
| 6. Sugar-sweetened beverages | 0 g /day | 1 |
|  | > 0 and < 250 g/day | 0.5 |
|  | ≥ 250 g/day | 0 |
| 7. Alcohol consumption | 0 g ethanol/day | 1 |
|  | > 0 and ≤ 20 g ethanol/day (women) > 0 and ≤ 40 g ethanol/day (men) | 0.5 |
|  | > 20 g ethanol/day (women) > 40 g ethanol/day (men) | 0 |
| 8. Smoking status | Never smoked | 1 |
|  | Used to smoke | 0.5 |
|  | Currently smoke | 0 |
| Healthy Lifestyle definitions were based on the 2018 WCRF/AICR recommendations.  HL: Healthy Lifestyle, BMI: Body Mass Index, MVPA: Moderate to Vigorous Physical Activity, RM: Red Meat, PM: Processed Meat | | |

Table S2. The mean value of metabolites was identified in the elastic net regression model using the HL score as the categorical or continuous variable (n=1833).

| **HL categories** | | **HL score** | |
| --- | --- | --- | --- |
| Metabolites | mean | Metabolites | mean |
| Cotinine | -0.255 | Cotinine | -0.107 |
| Caffeine | -0.127 | Hydroxycotinine | -0.077 |
| Urate | -0.111 | Caffeine | -0.047 |
| Hydroxycotinine | -0.110 | Glutamate | -0.034 |
| C5 carnitine | -0.065 | C54:1 TAG | -0.032 |
| C16:1 CE | -0.050 | Hydroxyproline | -0.031 |
| Hydroxyproline | -0.046 | C5 carnitine | -0.031 |
| C7 carnitine | -0.043 | C52:1 TAG | -0.029 |
| Arginine | -0.037 | Guanidoacetic acid | -0.026 |
| C54:1 TAG | -0.031 | Urate | -0.025 |
| Glutamate | -0.030 | Cyclohexylamine | -0.023 |
| C52:1 TAG | -0.022 | C16:1 CE | -0.020 |
| Isoleucine | -0.005 | Piperine | -0.017 |
| N-Methyl-L-proline | 0.011 | Uric acid | -0.012 |
| 4-Hydroxyhippurate | 0.015 | Isoleucine | -0.011 |
| C38:2 PE | 0.015 | C50:0 TAG | -0.011 |
| Cortisol | 0.016 | Metronidazole | -0.010 |
| C53:3 TAG | 0.030 | DMGV | -0.009 |
| 4-Pyridoxate | 0.040 | C36:2 PS plasmalogen | -0.009 |
| C22:0 LPE | 0.044 | Acetaminophen | -0.008 |
| Hexose monophosphate | 0.058 | 7-methylguanine | -0.007 |
| C36:3 PE | 0.073 | C7 carnitine | -0.007 |
| Indole-3-propionate | 0.079 | N-carbamoyl-beta-alanine | -0.005 |
| C22:5 CE | 0.191 | 1-Methylhistamine | -0.005 |
|  |  | C9 carnitine | -0.003 |
|  |  | Glycine | 0.002 |
|  |  | Cortisol | 0.003 |
|  |  | Thiamine | 0.003 |
|  |  | Thyroxine | 0.003 |
|  |  | C18:0 LPE | 0.004 |
|  |  | C20:0 LPE | 0.004 |
|  |  | Adipate | 0.004 |
|  |  | N-Methyl-L-proline | 0.005 |
|  |  | C36:4 PCA | 0.005 |
|  |  | Citrate | 0.005 |
|  |  | Hippurate | 0.007 |
|  |  | C2 carnitine | 0.007 |
|  |  | C38:6 PE | 0.007 |
|  |  | Proline betaine | 0.007 |
|  |  | C53:3 TAG | 0.009 |
|  |  | Xanthine | 0.009 |
|  |  | C51:3 TAG | 0.009 |
|  |  | Pyroglutamic acid | 0.009 |
|  |  | N-Acetylornithine | 0.010 |
|  |  | Glutamine | 0.012 |
|  |  | 4-Hydroxyhippurate | 0.012 |
|  |  | 4-Pyridoxate | 0.013 |
|  |  | C38:5 PE | 0.013 |
|  |  | Uracil | 0.014 |
|  |  | C38:2 PC | 0.015 |
|  |  | Malate | 0.017 |
|  |  | Uridine | 0.017 |
|  |  | Hexose monophosphate | 0.021 |
|  |  | C38:2 PE | 0.028 |
|  |  | Indole-3-propionate | 0.030 |
|  |  | C36:3 PE | 0.032 |
|  |  | C22:0 LPE | 0.034 |
|  |  | C22:5 CE | 0.040 |

Mean coefficient of metabolites in the elastic net regression model. The underlined metabolites were selected in both HL classification and HL score models using lambda.1se.

Table S3. Hazard ratio (95% CIs) for incident T2D and CVD using metabolomic signatures of HL in the PREDIMED population for each intervention group cohort using lambda.1se.

**Control group**

|  | Baseline visit | | 1-year visit | |
| --- | --- | --- | --- | --- |
|  | HR (95% CI) | *P* | HR (95% CI) | *P* |
| **Type 2 diabetes** | | | | |
| Incident cases/total participants | 88/306 |  | 60/226 |  |
| Model 1 | 0.80 (0.45, 1.25) | 0.280 | 1.17 (0.51, 2.66) | 0.712 |
| Model 2 | 0.81 (0.41, 1.37) | 0.348 | 1.13 (0.50, 2.53) | 0.771 |
| Model 3 | 0.80 (0.42, 1.52) | 0.496 | 1.15 (0.49, 2.73) | 0.744 |
| **Cardiovascular disease** | | | | |
| Incident cases/total participants | 77/307 |  | 51/274 |  |
| Model 1 | 0.83 (0.46, 1.50) | 0.534 | 1.07 (0.54, 2.16) | 0.831 |
| Model 2* | 0.83 (0.46, 1.49) | 0.528 | 1.08 (0.48, 2.47) | 0.838 |
| Model 3 | 0.86 (0.45, 1.64) | 0.645 | 1.30 (0.55, 3.03) | 0.544 |

**Mediterranean diet supplemented with extra virgin olive oil group**

|  | Baseline visit | | 1-year visit | |
| --- | --- | --- | --- | --- |
|  | HR (95% CI) | *P* | HR (95% CI) | *P* |
| **Type 2 diabetes** | | | | |
| Incident cases/total participants | 75/282 |  | 50/229 |  |
| Model 1 | 0.30 (0.17, 0.55) | <0.001 | 0.21 (0.09, 0.51) | <0.001 |
| Model 2 | 0.31 (0.16, 0.59) | <0.001 | 0.16 (0.06, 0.46) | <0.001 |
| Model 3 | 0.23 (0.10, 0.52) | <0.001 | 0.17 (0.06, 0.51) | 0.002 |
| **Cardiovascular disease** | | | | |
| Incident cases/total participants | 81/368 |  | 65/349 |  |
| Model 1 | 0.59 (0.34, 1.01) | 0.053 | 0.70 (0.42, 1.18) | 0.186 |
| Model 2* | 0.61 (0.34, 1.11) | 0.105 | 0.74 (0.39, 1.40) | 0.358 |
| Model 3 | 0.61 (0.33, 1.15) | 0.128 | 0.99 (0.51, 1.95) | 0.985 |

**Mediterranean diet supplemented with nuts group**

|  | Baseline visit | | 1-year visit | |
| --- | --- | --- | --- | --- |
|  | HR (95% CI) | *P* | HR (95% CI) | *P* |
| **Type 2 diabetes** | | | | |
| Incident cases/total participants | 82/335 |  | 51/250 |  |
| Model 1 | 0.47 (0.28, 0.78) | 0.004 | 0.50 (0.23, 1.10) | 0.085 |
| Model 2 | 0.37 (0.20, 0.66) | <0.001 | 0.14 (0.03, 0.58) | 0.007 |
| Model 3 | 0.44 (0.23, 0.80) | 0.007 | 0.19 (0.05, 0.74) | 0.017 |
| **Cardiovascular disease** | | | | |
| Incident cases/total participants | 64/318 |  | 64/318 |  |
| Model 1 | 0.58 (0.34, 0.99) | 0.049 | 0.57 (0.30, 1.12) | 0.101 |
| Model 2* | 0.45 (0.25, 0.78) | 0.005 | 0.38 (0.18, 0.78) | 0.009 |
| Model 3 | 0.36 (0.19, 0.66) | 0.001 | 0.38 (0.18, 0.82) | 0.014 |

Cox proportional hazard models with Barlow weights were used to estimate the HRs between HL categories.

Model 1: metabolite profile adjusted for age, sex and propensity scores and stratified by recruitment center and intervention group. Model 2: model 1 + education level + family history of CHD + dyslipidemia, lipid-lowering treatment, hypertension, and antihypertensive treatment. Model 3: model 2 + self-reported HL score (as categorical or continuous).

*Model 2 of the CVD-case cohort was further adjusted for diabetes prevalence.

HL, healthy lifestyle; HR, hazard ratio; CI, confidence interval; P, p value.


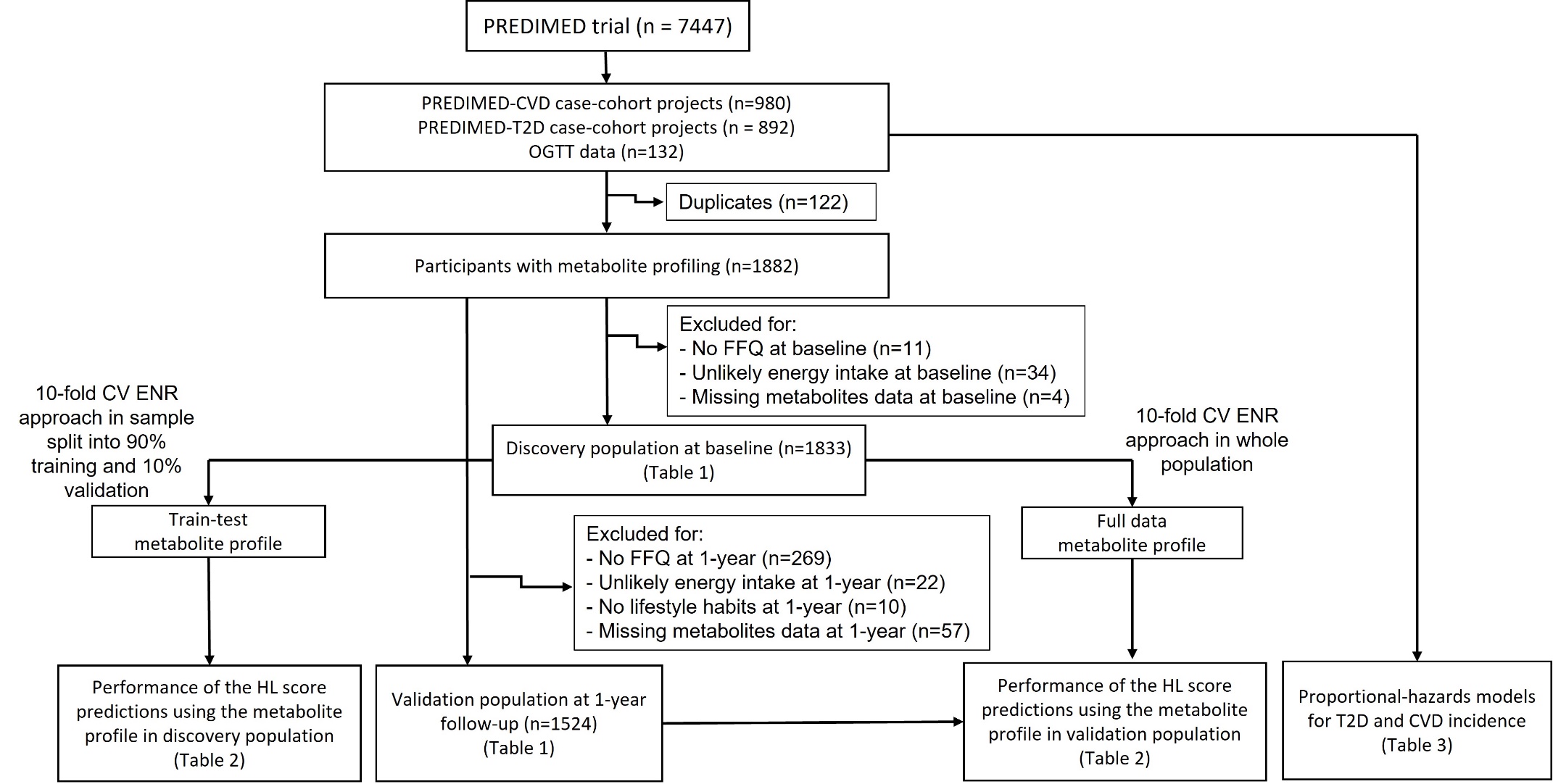


Figure S1. Flow-chart of participants and analysis steps. HL, Healthy Lifestyle; FFQ, Food Frequency Questionnaire; OGTT, Oral Glucose Tolerance Test; CV, Cross-Validation; ENR, Elastic Net Regression.


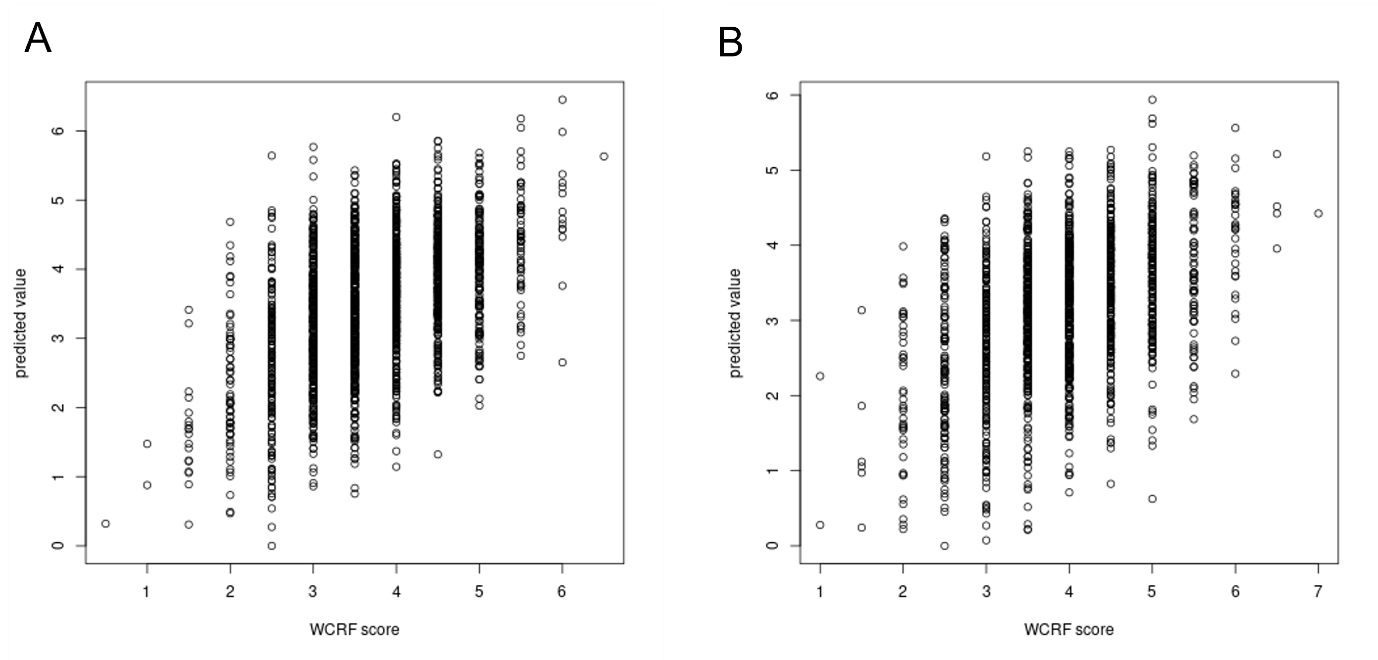


Figure S2. Scatterplot of the correlation between the HL score and the predicted values of the HL score using the metabolite profile at baseline (discovery) (A) and 1-year follow-up (validation) (B) data sets.


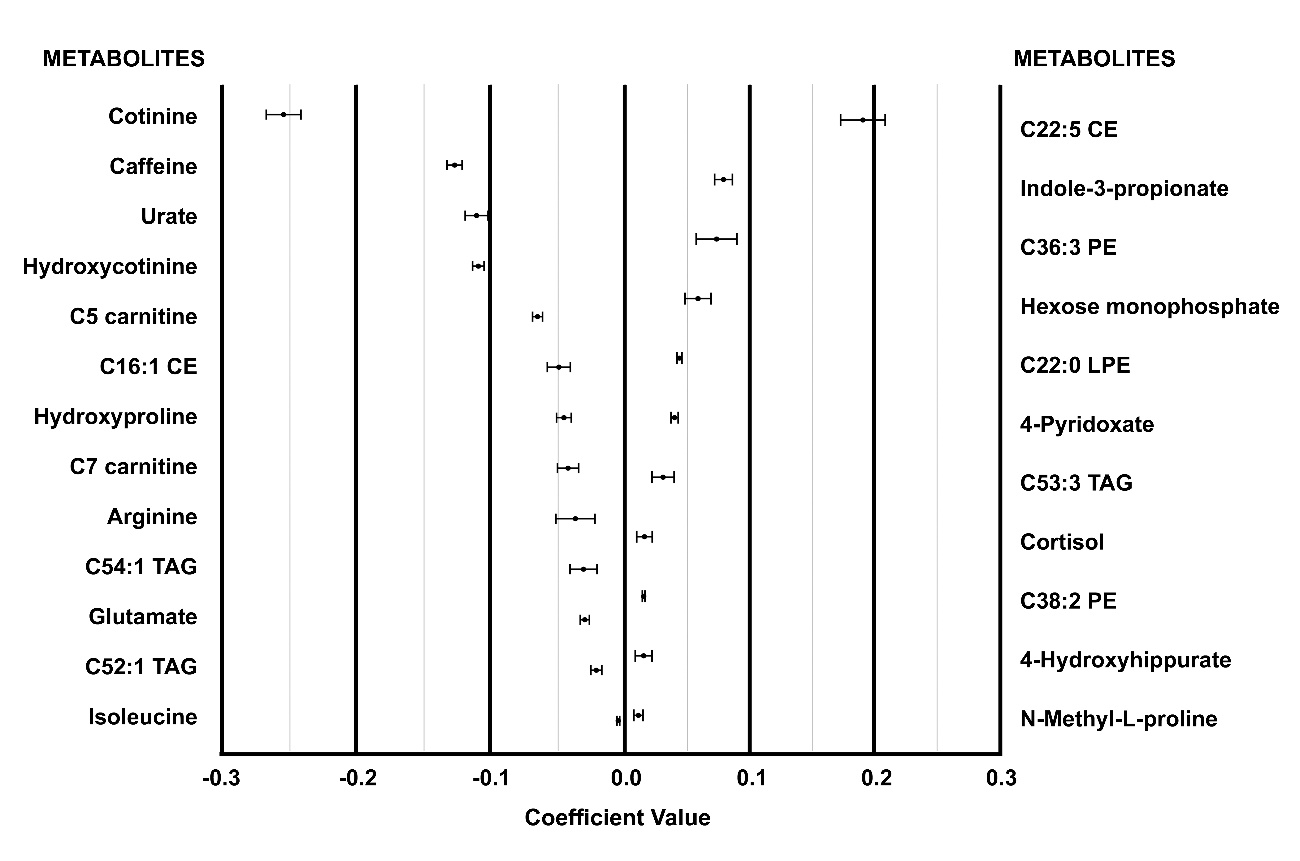


Figure S3. Logistic regression coefficients (mean and SD) of the 24 metabolites selected using the HL score categorical variable. Metabolites were selected ten times in the 10-cross-validation elastic net regression in the whole dataset (n=1833). Metabolites with negative coefficients (n = 13) are plotted on the left-hand side, whereas those with positive coefficients (n = 11) are plotted on the right-hand side.
